# Global mRNA expression analysis in myosin II deficient strains of *Saccharomyces cerevisiae *reveals an impairment of cell integrity functions

**DOI:** 10.1186/1471-2164-9-34

**Published:** 2008-01-23

**Authors:** José F Rodríguez-Quiñones, Rafael A Irizarry, Nitza L Díaz-Blanco, Félix E Rivera-Molina, Diana Gómez-Garzón, José R Rodríguez-Medina

**Affiliations:** 1 Department of Biochemistry, School of Medicine, Medical Sciences Campus, University of Puerto Rico, P.O. Box 365067, San Juan, Puerto Rico 00936-5067, USA; 2 Department of Biostatistics, Bloomberg School of Public Health, Johns Hopkins University, 615 N. Wolfe St. E3620, Baltimore, MD 21205, USA; 3 Department of Biology, University of Puerto Rico at Bayamón, 170 Carr. 174 Urb. Ind. Minillas, Bayamón, Puerto Rico 00959, USA; 4 Department of Cell Biology, School of Medicine, Yale University SHM C-232, New Haven, CT 06520, USA; 5 Department of Science and Technology, Universidad Metropolitana, P.O. Box 21150, San Juan, Puerto Rico 00928, USA

## Abstract

**Background:**

The *Saccharomyces cerevisiae MYO1 *gene encodes the myosin II heavy chain (Myo1p), a protein required for normal cytokinesis in budding yeast. Myo1p deficiency in yeast (*myo1Δ*) causes a cell separation defect characterized by the formation of attached cells, yet it also causes abnormal budding patterns, formation of enlarged and elongated cells, increased osmotic sensitivity, delocalized chitin deposition, increased chitin synthesis, and hypersensitivity to the chitin synthase III inhibitor Nikkomycin Z. To determine how differential expression of genes is related to these diverse cell wall phenotypes, we analyzed the global mRNA expression profile of *myo1Δ *strains.

**Results:**

Global mRNA expression profiles of *myo1Δ *strains and their corresponding wild type controls were obtained by hybridization to yeast oligonucleotide microarrays. Results for selected genes were confirmed by real time RT-PCR. A total of 547 differentially expressed genes (p ≤ 0.01) were identified with 263 up regulated and 284 down regulated genes in the *myo1Δ *strains. Gene set enrichment analysis revealed the significant over-representation of genes in the protein biosynthesis and stress response categories. The *SLT2/MPK1 *gene was up regulated in the microarray, and a *myo1Δslt2Δ *double mutant was non-viable. Overexpression of ribosomal protein genes *RPL30 *and *RPS31 *suppressed the hypersensitivity to Nikkomycin Z and increased the levels of phosphorylated Slt2p in *myo1Δ *strains. Increased levels of phosphorylated Slt2p were also observed in wild type strains under these conditions.

**Conclusion:**

Following this analysis of global mRNA expression in yeast *myo1Δ *strains, we conclude that 547 genes were differentially regulated in *myo1Δ *strains and that the stress response and protein biosynthesis gene categories were coordinately regulated in this mutant. The *SLT2/MPK1 *gene was confirmed to be essential for *myo1Δ *strain viability, supporting that the up regulated stress response genes are regulated by the *PKC1 *cell integrity pathway. Suppression of Nikkomycin Z hypersensitivity together with Slt2p phosphorylation was caused by the overexpression of ribosomal protein genes *RPL30 *and *RPS31*. These ribosomal protein mRNAs were down regulated in the *myo1Δ *arrays, suggesting that down regulation of ribosomal biogenesis may affect cell integrity in *myo1Δ *strains.

## Background

In yeast cells as in animal cells, cytokinesis requires the formation of an actomyosin contractile ring [1]. *Saccharomyces cerevisiae *possesses a single *MYO1 *gene, which encodes the myosin II heavy chain protein (Myo1p), the only conventional myosin II of the budding yeast. Myo1p is associated with F-actin during formation of the contractile apparatus, and provides the actin-activated ATPase activity driving the contraction of the actomyosin ring at cytokinesis [2,3]. Previous studies have described the importance of Myo1p in cytokinesis in yeast by establishing that myosin II concentrates at the bud neck, contracts during the cytokinesis, and subsequently is redistributed or degraded after the completion of mitosis [2,4].

In addition to the loss of normal cytokinesis function, *myo1Δ *strains present phenotypes such as the formation of attached cells, loss of axial budding pattern, cell enlargement, osmotic hypersensitivity, delocalized chitin deposition [5], increased chitin synthesis[6,7], and hypersensitivity to Nikkomycin Z [8]. The diversity of these phenotypes, many of which can be associated with cell wall organization, suggests that myosin II may affect cellular functions other than cytokinesis. The purpose of this study was to generate a global mRNA expression profile of *myo1Δ *strains using yeast oligonucleotide microarrays to identify potential relationships between the multiple *myo1Δ *phenotypes mentioned and changes in the transcription profile of specific genes that can be functionally related to these phenotypes.

## Results and Discussion

The results of independent microarray hybridization experiments conducted with two different *myo1Δ *knockout strains were averaged and combined for comparison with the corresponding wild-type control values. Our rationale for this experimental design was to identify deferentially regulated genes held in common between at least two different *myo1Δ *strain backgrounds in order to eliminate any strain specific differences and preserve strictly *myo1Δ*-dependent genetic phenotypes. Cells were cultured in Histidine dropout medium or CSM to maintain the culture conditions under which *myo1Δ *strains have been shown to require chitin synthase III expression for their survival [8] as well as to maintain equivalent growth conditions between mutant and wild-type strains, respectively.

### Up regulated genes in *myo1Δ*

Previously published studies reported differentially expressed genes under conditions such as cell wall mutations, chemical and biochemical perturbations of the cell wall [9], diverse mutations covering 4% of the yeast genome [10], and during the cell cycle [11], employing values with p ≤ 0.05. We identified 97 genes in common with these previous studies out of a total of 2,273 genes with p ≤ 0.05 that were differentially expressed in myo1Δ strains (data not shown).

To refine this analysis we used a more stringent cutoff (p ≤ 0.01) to identify 547 differentially expressed genes in *myo1Δ *strains. We observed a total of 263 up regulated genes in *myo1Δ *strains compared to the wild type control strains. The functional categories of the up regulated genes that were represented as listed in the *Saccharomyces* Genome Database (SGD) were: cell organization and biogenesis (17/263), metabolism (25/263), transport (9/263), carbohydrate metabolism (23/263), stress response (17/263), protein biosynthesis (2/263), cell cycle (3/263), signal transduction (7/263), transcription (1/263), sporulation (4/263), budding (4/263), RNA processing (4/263), protein transport (6/263), protein amino acid phosphorylation (2/263), protein amino acid dephosphorylation (1/263), autophagy (2/263), DNA repair (5/263), cell growth and maintenance (1/263), and unknown functions (130/263) (Additional file 1).

The results of this DNA microarrays analysis were validated by real time RT-PCR using a subset of these up regulated genes. The real time RT-PCR results indicated a fold change of 1.8, 3.5, 13.0, 3.8, 3.5 and 2.3 for *SLT2, ECM4, SPI1, YHR097C, ROM1 *and *IRA2 *respectively that were consistent with the microarray results (Table 1).

**Table 1:** Validation of DNA microarray results. mRNAs were selected for real time RT-PCR analysis from the list of differentially expressed genes in *myo1*Δ with p < 0.01(Additional File 1). Fold-change was estimated by a semi-quantitative method as described in the Methods section.

Gene name	Fold Change in Microarray	Fold Change (real time RT-PCR)
Up regulated genes		
*SLT2*	2.1	1.8
*ECM4*	3.2	3.5
*SPI1*	8.6	13.0
*YHR097C*	4.1	3.8
*ROM1*	3.2	3.5
*IRA2*	3.7	2.3
Down regulated genes		
*RPL30*	3.1	6.3
*RPS31*	2.6	7.7

### Down regulated genes in *myo1Δ*

A total of 284 genes were down regulated in the *myo1Δ *strains. As was expected for the knockout mutation, the *MYO1 *gene was identified among the down regulated genes in *myo1Δ *strains (4.5-fold, Additional file 1). Represented functional categories established by the SGD were: cell organization and biogenesis (59/284), transport (16/284), protein biosynthesis (72/284), metabolism (39/284), DNA metabolism (12/284), carbohydrate metabolism (7/284), stress response (2/284), cell cycle (5/284), signal transduction (1/284), transcription (5/284), sporulation (2/284), budding (5/284), cell growth and maintenance (1/284), protein transport (4/284), DNA damage response (1/284), RNA localization (1/284), protein amino acid phosphorylation (1/284), RNA processing (5/284), and genes of unknown function (46/284) (Additional File 1). Real time RT-PCR results for *RPL30 *and *RPS31 *indicated negative fold changes of 6.3 and 7.7 respectively that were consistent with the microarray results (Table 1).

### Gene Set Enrichment Analysis

**Figure 1:**
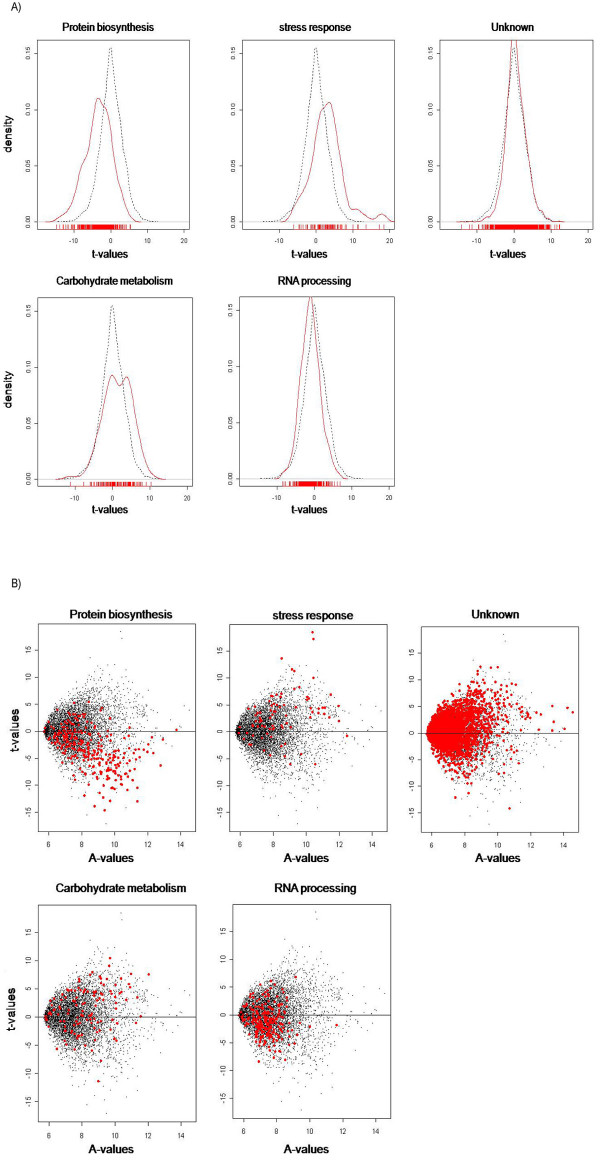
Histograms derived from Gene Set Enrichment Analysis for categories with a corrected p-value ≤ 0.0004. A) Density versus t-value plots for protein biogenesis, stress response, unknown, carbohydrate metabolism, and RNA processing categories. Red lines represent the distribution of genes of a specific category in the array. Black lines represent the distribution of all genes in the array. B) t-value versus A-value plots for protein biogenesis, stress response, unknown, carbohydrate metabolism, and RNA processing categories. Red dots represent the genes of a specific category in the array. Black dots represent the distribution of all genes in the array. The cutoff for a significant category is based on a p-value calculated after 10,000 permutations and then a corrected p-value was calculated using the Bonferroni correction. See Methods section for details.

In order to identify functionally related gene sets that were differentially expressed and behaved in a coordinated fashion in the *myo1Δ *strains, a Gene Set Enrichment Analysis (GSEA) [12] was performed. This kind of analysis is useful for whole genome expression assays, because it allows the visualization of the data in groups and how these groups are represented in the microarray. For this analysis a gene set was created for all the 6,256 genes contained in the array, considering the t-value for each gene, and then calculating the mean of the t-values for each category. The genes were classified in categories by biological function according to the *Saccharomyces *genome database (SGD) [13]. The GSEA analysis was performed using the Limma package from Bioconductor [14] as described in the materials and methods section. Of the 25 categories considered for GSEA, there were five categories with a corrected p-value below the cutoff (p-value ≤ 0.0004). These categories were: protein biosynthesis, stress response, RNA processing, carbohydrate metabolism and genes of unknown function. Histograms of density versus t-value were generated for each category to observe the distribution of genes of a specific category compared with the normal distribution of all the categories on the arrays (Figure 1A.). Plots of t-values vs. A-values were created to identify the genes in each category and observe their distribution across the array (Figure 1B). Of the five gene categories selected, the protein biosynthesis and stress response categories presented the most dramatic changes in their normal distribution. Although the other three categories had p-values below the cutoff, when we observe their respective histograms there were less dramatic differences in the distribution of each group compared to the whole microarray. The protein biosynthesis category is interesting because the histogram shows a shift from the normal distribution towards negative t-values (Figure 1A). This is also observed in the corresponding t-value vs. A-value plot where we can see a greater quantity of genes biased towards negative t-values (Figure 1B). This result was predictable due to the great quantity of genes related to protein biosynthesis that were down regulated in the microarray. Within the protein biosynthesis gene category there were an unusually high number of ribosomal protein genes that were down regulated suggesting that ribosome biogenesis may be affected in the *myo1Δ *strains. This interpretation would be consistent with the relatively slow growth rate reported for these cells compared to their wild-type controls [5]. Conversely, in the histogram for stress response genes, there was a shift from the normal distribution of the array towards positive t-values (Figure 1A). This shift can be explained by the up-regulation of stress response genes with high t-values, therefore causing this type of shift to the right of the normal distribution curve. Considering the t-value vs. A-value plot, the stress response genes were distributed above zero towards positives t-values. Among these stress response genes were *GRE1 *and *GRE3 *that are regulated by the High Osmolarity Glycerol response-signaling pathway (*HOG*) [15]. Another stress response gene that had the highest t-value was *HOR7*. This gene encodes a putative GPI anchored cell wall protein of unknown function that was also previously reported to be related to high osmolarity response [15]. Differential expression of these genes indicates that genes in this pathway may become activated in *myo1Δ *strains despite the absence of hyperosmotic conditions in our experimental design. However, a *hog1Δ *deletion was viable in these *myo1Δ *strains indicating that this pathway is not essential for *myo1Δ *cell viability under our culture conditions (data not shown).

Although the "unknown function" category had a greater representation of differentially expressed genes in the array, there was no shift in the density vs. t-values histogram (Figure 1A) or the t-values vs. A-values plot (Figure 1B). Due to the fact that this category also has more genes represented in the yeast genome, this group was not considered of interest for this analysis. However, we are aware that this category represents the majority population of differentially expressed genes many of which exhibit extraordinary fold-changes in the double-digit range. These could represent a pool of genes with potentially novel functions related to cell proliferation, cell division, and cell wall organization.

Carbohydrate metabolism was another functional category exhibiting a large number of genes with dramatic ranges in mRNA expression levels. The GSEA results for this category showed a bimodal distribution (Figure 1A). However, in the t-value vs. A-value plot these genes were distributed throughout the array with no apparent bias towards negative or positive t-values (Figure 1B). This bimodal distribution may be significant as it may represent the existence of two sub-populations of genes with distinct behaviors.

### Differential regulation of Pkc1p/Slt2p cell integrity pathway

**Figure 2:**
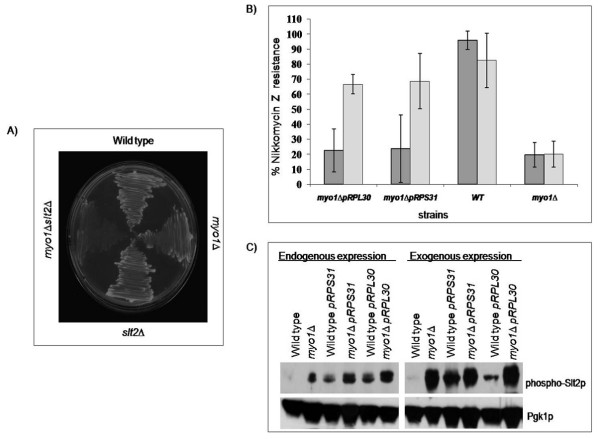
(A) Genetic disruption of the *SLT2/MPK1 *gene induces lethality in a *myo1Δ *strain. Wild type, *myo1Δ*, *slt2Δ *and *myo1Δ slt2Δ*pRS316-*MYO1 *strains were grown in CSM (1 mg/ml) 5-FOA for three days at 26°C. (B)Suppression of Nikkomycin Z hypersensitivity in *myo1Δ *strains overexpressing the ribosomal protein genes *RPL30 *and *RPS31*. Wt, *myo1Δ, myo1Δ*pRS316-*RPL30 *and *myo1Δ*pRS316-*RPS31 *strains were grown CSM or CSM *URA*^- ^in presence or absence of 6.25 μM Nikkomycin Z in 2% glucose or 2% galactose for 48 hours at 26°C. Percent of Nikkomycin Z resistance was calculated as: (OD_600 nm treated_/OD_600nm untreated_) × (100). Dark gray histograms represent cells where expression of the plasmid is repressed with 2% glucose and light gray histograms represent cells where expression of the plasmid gene is induced with 2% galactose. (C) Determination of the activation of the cell integrity pathway in *myo1Δ *strains overexpressing the ribosomal protein genes *RPL30 *and *RPS31*. Wild type, *myo1Δ*, wildtype pRS316-*RPL30*, wild type pRS316-*RPS31, myo1Δ*pRS316-*RPL30 *and *myo1Δ*pRS316-*RPS31 *strains were grown in CSM or CSM *URA*^- ^in the presence of 2%glucose or 2% galactose. Equal amounts of protein (75 μg) were analyzed by Western blot as described in the Methods section. The phosphorylated levels of Slt2p were observed using a mouse monoclonal antibody against phospho-p42/p44 Slt2p (p-Slt2p). The membrane was stripped and reprobed with a monoclonal antibody against Pgk1p as a loading control.

The *SLT2/MPK1*gene that encodes a serine/threonine MAP kinase involved in regulating the maintenance of cell integrity [9,16-18] was up regulated approximately 2-fold in the *myo1Δ *strains (Table 1 and Additional file 1). In other studies it was reported that phospho-Slt2p was also activated in the *myo1Δ *mutant compared to the wild type strain [19,20]. A previous study defined a cluster of 20 genes regulated by this *PKC1*-mediated signaling pathway that were strongly induced by chemical perturbation of the cell wall with Congo red dye, by enzymatic treatment with Zymolyase, or by mutations in specific cell wall genes such as *KRE6*, *GAS1*, *FKS1, MNN9 *and *KNR4 *[9]. *SED1, HSP12, SLT2, FBP26 *and *YHR097C *(Additional file 1) represent five of these 20 genes that were also up regulated in the *myo1Δ *strains (p ≤ 0.01) further supporting that the *PKC1 *signaling pathway is activated. A *myo1Δslt2Δ *strain expressing the *MYO1 *gene on a pRS316 plasmid was grown at 26°C in 5-FOA to extract the plasmid and generate the *myo1Δslt2Δ *double knockout. After three days, no growth was observed for the *myo1Δslt2Δ *double knockout (Figure 2A). This result corroborates that *slt2Δ *is synthetically lethal in a *myo1Δ *mutant and supports that the cell integrity pathway is essential for viability in these strains [20].

### Suppression of Nikkomycin Z hypersensitivity by overexpression of ribosomal protein genes

Gene Set Enrichment Analysis showed that protein biosynthesis genes were coordinately down regulated in these *myo1Δ *strains (Figure 1B). *RPL30 *and *RPS31 *ribosomal protein genes were among those differentially expressed in the *myo1Δ *microarrays. The *RPL30 *gene is essential in budding yeast [21] and its protein product is a component of the large ribosomal (60S) subunit. The *RPS31 *gene product constitutes a fusion of a non-essential small ribosomal (40S) subunit protein with ubiquitin, which facilitates the assembly of ribosomal proteins into ribosomes and is required for the proper translation of *GCN4*, a transcription factor that regulates amino acid biosynthesis [22]. A previous screen for suppressors of Nikkomycin Z (NZ) hypersensitivity in *myo1Δ *strains identified the cDNAs encoding *RPL30 *and *RPS31 *among others [24]. Nikkomycin Z is a competitive inhibitor of Chs3p and our previous studies established the hypersensitivity of *myo1Δ *strains to this antibiotic which inhibits cell growth and affects cell integrity by blocking chitin synthesis [8]. Recombinant *RPL30 *and *RPS31 *conferred 67% and 69% resistance to Nikkomycin Z respectively, when overexpressed by induction with 2% galactose (Figure 2B). We confirmed increased transcription of both ribosomal protein cDNAs in a wild-type strain by real time RT-PCR (9.13 and 8.03 respectively). These results support that overexpression of ribosomal protein genes may suppresses NZ hypersensitivity in *myo1Δ *strains by contributing to restore cell integrity. Though the mechanism is unclear, other RP genes identified in our previous study had a similar effect.

### Western blot analysis of the level of phosphorylated Slt2p in *myo1Δ *strains overexpressing *RPL30 *or *RPS31*

To relate suppression of NZ hypersensitivity with the restoration of cell integrity in *myo1Δ *strains, we assayed the level of phosphorylated Slt2p (p-Slt2p) by Western blot. It has been previously established that the cell integrity pathway is activated in *myo1Δ *mutants [19,20]. Our results show increases in p-Slt2p levels for *myo1Δ *strains overexpressing *RPL30 (myo1Δ*pRS316-*RPL30*) and *RPS31 *(*myo1Δ*pRS316-*RPS31*) (Figure 2C). Moreover, we also detected increases in p-Slt2p levels for wild type strains overexpressing *RPL30 *(wild type pRS316-*RPL30*) and *RPS31 *(wild type pRS316-*RPS31*) genes in repressing and inducing conditions (Figure 2C). The former observation can be explained by leakage from the *GAL1 *promoter under repressing conditions (data not shown) and suggested that the activation of the cell integrity pathway can respond to very minor changes in RP gene transcription. When *SLT2 *mRNA levels were measured, the fold changes for strains wild type pRS316-*RPL30*, wild type pRS316-*RPS31*, *myo1Δ*pRS316-*RPL30*, and *myo1Δ*pRS316-*RPS31 *were 1.21, 2.73, 1.6 and 6.5, respectively (see Table 2). While both RP cDNAs had similar effects on p-Slt2p levels, fold changes in *SLT2 *mRNA levels induced by RP overexpression were consistently lower for *RPL30 *than for *RPS31*.

**Table 2:** Determination of the *SLT2 *gene mRNA fold change for wild type pRS316-*RPL30*, wild type pRS316-*RPS31*, *myo1Δ*pRS316-*RPL30*, and *myo1Δ*pRS316-*RPS31 *strains grown under inducing (2% galactose) and repressing (2% glucose) conditions.

Strains	*SLT2 *gene fold change
wild type pRS316-*RPL30*	1.21
wild type pRS316-*RPS31*	2.73
*myo1Δ *pRS316-*RPL30*	1.6
*myo1Δ *pRS316-*RPS31*	6.5

## Conclusion

Following our analysis of the global mRNA expression profile in *myo1Δ *strains of *Saccharomyces cerevisiae *we found that there were 547 differentially regulated genes. Gene Set Enrichment Analysis indicated that stress response and protein biosynthesis gene categories are inversely related in this mutant. These results support that cell wall morphogenetic processes were affected by myosin II deficiency. The up-regulation of stress response genes in the *myo1Δ *strains reflects the essentiality of the cell integrity pathway and the importance of the *PKC1*-mediated signaling pathway for survival in these strains. The coordinated down-regulation of protein biosynthesis genes can be related to a loss of cell wall integrity in this mutant because overexpression of *RPL30 *and *RPS31*, two differentially regulated ribosomal protein (RP) genes, conferred Nikkomycin Z resistance to the hypersensitive *myo1Δ *strains. Overexpression of *RPL30 *and *RPS31 *did not shut down the cell integrity pathway supporting that their overexpression promotes activation of the *PKC1 *pathway.

The high frequency of ribosomal protein genes in the down regulated gene category implies that ribosome biogenesis is greatly affected by myosin type II deficiency. It has been proposed that yeast cells regulate ribosome biogenesis primarily by responding to growth limiting environmental signals [23]. Cell wall and plasma membrane stress may therefore represent such signals that can activate signaling pathways for regulating growth rate in the absence of myosin II. We propose that this alteration in the normal regulation of these processes may be necessary for the completion of cytokinesis in the absence of a contractile ring that must involve increased chitin synthesis and extensive cell wall remodeling at the bud neck.

We previously demonstrated that overexpression of the cDNA for ubiquitin conjugating enzyme *UBC4 *lowered the p-Slt2p levels and conferred resistance to NZ in *myo1Δ *strains, suggesting that cell integrity was restored [24]. We have now discovered that overexpression of *RPL30 *and *RPS31 *can also up regulate the cell integrity pathway to achieve a similar effect. It is unclear how such an alteration in the level of transcription of individual RP genes can affect this pathway but it is known that ribosome biosynthesis is a tightly regulated process in yeast that can be affected by altering the stoichiometry of the individual ribosomal subunits [25]. This observation could therefore represent an effect of up regulation of ribosome biosynthesis on cell integrity. Alternatively, the response of the *PKC1 *pathway to changes in RP gene transcription may represent one aspect of a novel feedback regulatory loop. Others have reported that inhibition of the *TOR *pathway by rapamycin treatment activated the *PKC1 *cell integrity pathway in yeast cells [26]. It was acknowledged that inhibition of the *TOR *pathway also results in repression of the ribosomal biogenesis process [26]. The similarity of the described phenotypes with those of *myo1Δ *strains merits further investigation to determine if down regulation of the *TOR *pathway is a contributing factor to the phenotypes in these strains.

## Methods

### Strains and culture conditions

All the experiments were performed using *Saccharomyces cerevisiae *wild type and *myo1Δ *strains listed in Table 3. YJR6 is a *myo1Δ ::HIS5 *strain generated by homologous recombination in the parental haploid wild type strain, MGD-353-46D using a PCR based method. YJR12 (wild type) and YJR13 (*myo1Δ*) strains were obtained as haploid segregants from a cross between YJR6 and BY4741 (obtained from ATCC). Cultures were grown overnight at 26°C to an optical density between 0.5–0.8 (OD_600_) in complete synthetic media (CSM, 2% glucose, 1× Nitrogen base) or Histidine dropout media (CSM-HIS^-^) with continuous shaking at 200 rpm. Where indicated in Table 3, *myo1Δ *strains were transformed with plasmids containing cDNAs for *RPL30 *(pRS316-*RPL30*) or *RPS31 *(pRS316-*RPS31*) regulated by the *GAL1 *promoter, or *MYO1 *(pRS316-*MYO1*) regulated by its natural promoter.

**Table 3:** Strains used in this study.

Strain	Genotype	Source
BY4741	MAT a *his3delta1 leu2delta0 met15delta0 ura3delta0*	ATCC
YJR12 (wild type)	MAT α *trp1 ura3 leu2-3 his3delta1 *ADE^+ ^ARG^+ ^*cyh*^*R*^	Lab. strain
YJR13 (*myo1Δ*)	MAT a *trp1 ura3 leu2-3 his3delta1 *ADE^+ ^ARG *cyh*^*R*^*myo1delta::HIS5*^+^	Lab. strain
MGD353-46D (wild type)	MAT α *trp1-289 ura3-52 leu2-3, 112 his3delta1 *ADE^+ ^ARG *cyh*^*R*^	B. Rymond
YJR6 (*myo1Δ*)	MAT α *trp1-289 ura3-52 leu2-3, 112 his3delta1 *ADE^+ ^ARG *cyh*^*R *^*myo1delta::HIS5*^+ ^parental MGD353-46D	F. Rivera
YJR13 pRS316-*RPL30 *(*myo1Δ *pRS316-*RPL30*)	MAT a *trp1 ura3 leu2-3 his3delta1 *ADE^+ ^ARG *cyh*^*R*^*myo1delta::HIS5*^+^, pRS316-*RPL30*	A. Brestcher (plasmid)
YJR13 pRS316-*RPS31 *(*myo1Δ *pRS316-*RPS31*)	MAT a *trp1 ura3 leu2-3 his3delta1 *ADE^+ ^ARG *cyh*^*R*^*myo1delta::HIS5*^+, ^pRS316-*RPS31*	A. Brestcher (plasmid)
YJR13 pRS316-*MYO1 *(*myo1Δ *pRS316-*MYO1*)	MAT a *trp1 ura3 leu2-3 his3delta1 *ADE^+ ^ARG *cyh*^*R*^*myo1delta::HIS5*^+^, pRS316-*MYO1*	F. Rivera (plasmid)
YJR12 pRS316-*RPL30 *(wild type pRS316-*RPL30*)	MAT α *trp1 ura3 leu2-3 his3delta1 *ADE^+ ^ARG^+ ^*cyh*^*R *^pRS316-*RPL30*	A. Brestcher (plasmid)
YJR12 pRS316-*RPS31 *(wild type pRS316-*RPS31*)	MAT α *trp1 ura3 leu2-3 his3delta1 *ADE^+ ^ARG^+ ^*cyh*^*R *^pRS316-*RPS31*	A. Brestcher (plasmid)
YJF1 (*myo1Δslt2Δ *pRS316-*MYO1*)	MAT a *trp1 ura3 leu2-3 his3delta1 *ADE^+ ^ARG *cyh*^*R*^*myo1delta::HIS5*^+^, pRS316-*MYO1*, *slt2delta::KAN*^*R*^	This study

### RNA extraction procedure

Total RNA was extracted from 4 × 10^7 ^cells derived from triplicate biological replicate cultures of strains MGD353-46D, YJR6, YJR12, and YJR13 using the RNeasy Mini Kit for isolation of total RNA (Qiagen, Hilden, Germany) following manufacturer's instructions. RNA concentrations were determined by measuring absorbance at 260 nm using a Nanodrop spectrophotometer (Nanodrop Technologies, Wilmington, DE). The purity and integrity of the RNA was monitored using an Agilent Bioanalyzer (Agilent Technologies, Palo Alto, CA) following manufacturer's instructions.

### DNA microarrays hybridization and analysis

1.0 μg of total RNA from each sample was amplified using the Low RNA Input Fluorescent Linear Amplification kit (Agilent Technologies, Palo Alto, CA). The amplified cRNA was labeled with 10 mM Cyanine 5-CTP (Cy5) or Cyanine 3-CTP (Cy3) (Perkin Elmer Life Sciences, Boston, MA). Labeled cRNA's were purified with Qiagen RNeasy mini spin columns and dye incorporation was monitored on an Agilent Bioanalyzer. Hybridization of Cy5 and Cy3 labeled cRNA's were performed using Yeast Oligo Microarray slides and hybridization kit from Agilent Technologies in 1012AG hybridization chambers (Sheldon Manufacturing, Cornelius, Oregon) at 60°C overnight. Slides were washed at high stringency and scanned with a VersArray Chip Reader system (BioRad, Hercules, CA) at a resolution of 5 μm with detector sensitivity values between 704–800 and laser power at 85%. Scanned images were transferred to the Imagene 3.0 software program (Biodiscovery, El Segundo, CA) for further analysis to locate spots, adjust the appropriate grid, and obtain the Cy3 and Cy5 TIFF files. The microarrays raw data generated with Imagene 3.0 were analyzed using Limma software (Bioconductor Package 1.7). The data was prepared for analysis by correcting for background intensity. The individual data sets were normalized using the locally weighted linear regression (Lowess) within each array. After normalization, the difference between the experimental and control signal was calculated, replicates were combined, and their averages were calculated. The fold change in gene expression was calculated by 2^(M)^, where M is the log_2_-fold change after background correction and normalization. An Empirical Bayes Statistics for differential expression analysis [27] (eBayes statistics) was performed by Limma. Genes with a p-value ≤ 0.01 were established as a cutoff for differential expression. In addition, a false discovery rate (FDR) test [28] was performed by Limma [14]. The microarray raw data and the processed data are available at Gene Expression Omnibus of NCBI (GSE5931) [29].

### Gene Set Enrichment Analysis

Gene Set Enrichment Analysis was performed using the Limma package of Bioconductor [14]. For this purpose a gene set file was created classifying all the genes included in the microarray into groups according to their specific involvement in a biological process. From this gene set, a total of 25 categories were represented according to the Osprey network visualization software [30], matching the category with the t-value. Then, the Limma software calculated the average of the t-value for each category or biological process. Significance of differential expression as determined by the enrichment analysis was recalculated 10,000 times. A corrected p-value was obtained from the analysis using the Bonferroni correction. The initial cutoff to determine that the gene set was differentially expressed was p-value ≤ 0.01. Based on this correction, the cutoff for significance was established at a p-value ≤ 0.0004.

### Confirmation of microarray data by real time RT-PCR

Real time RT-PCR assays were performed with 30 ng of total RNA using the Quantitec SYBR Green RT-PCR kit (Qiagen, Valencia, CA) with primers at 0.5 μM (Table 4) and a final reaction volume of 25.0 μL, following manufacturer recommendations. The reactions were performed following the conditions recommended by the manufacturer using the iCycler iQ Multicolor real time PCR detection system (BioRad, Hercules, CA). The PCR quantification and melting curves were generated using the iCycler new version software (BioRad, Hercules, CA). The sequences of the forward and reverse primers for the selected mRNAs are listed in Table 4. The fold change was determined by the 2^ΔΔCt ^method [31]. The ΔC_t _of the control and experimental samples was calculated from the threshold cycle of the target gene minus the threshold cycle of the reference gene (*ACT1*). The ΔΔC_t _was calculated by subtracting the ΔC_t _of the control sample minus ΔC_t _of the experimental sample.

**Table 4:** Primers used for real time RT-PCR in this study.

Target	Forward primer	Reverse primer
*ACT1*	5'-GCCATTTTGAGAATCGATTTG-3'	5'-TTAGAAACACTTGTGGTGAAC-3'
*ECM4*	5'-GTGGTACAAACGGAGCTTTCA-3'	5'-GTGCCCAATGGACTACGCTACA-3'
*SPI1*	5'-CCAGAACCAACGACTTTCGTA-3'	5'-ACTGCACCAGCCAAACCTA-3'
*ROM1*	5'-AGCTATCTACGCCTCCAACT-3'	5'-ATGATGACGTTGGTGTTGA-3'
*SLT2*	5'-AGCAACAGCAGCCTTCAGA-3'	5'-GAACGCGAGGAAGTATCCAA-3'
*YHR097C*	5'-CCATCGTCGTACATCACAC-3'	5'-GTACAGGCGCCACTTTATTA-3'
*IRA2*	*5'-*ACTCACCTTTCCGCTGAC-3'	5'- CATGACACATCGCTTCTACA-3'
*RPL30*	5'-GATCATCATTGCCGCTAACA-3'	5'-GAGACAACACCGACTCTGAATAACT-3
*RPS31*	5'-ACAAGGAAGGTATTCCACCTGA-3'	5'-TCTTTCTCTTCTTACCACCACCTC-3'

### Nikkomycin Z sensitivity assay in *myo1Δ *strains overexpressing ribosomal protein genes

The screening for Nikkomycin Z sensitivity suppression in *myo1Δ *was performed as described in [24]. Briefly, 1 × 10^5 ^cells/ml of WT, *myo1Δ*, *myo1Δ*p *RPL30 *and *myo1Δ*p *RPS31 *cells were grown in ura^-^glu^- ^or ura^-^gal^- ^in the presence or absence of 6.25 μM Nikkomycin Z at 26°C and 225 rpm. The OD_600 nm _was determined after 48 hrs of growth. The % Nikkomycin Z resistance represents: (OD_600 nm treated_/OD_600 nm untreated_) × 100.

### *SLT2 *gene disruption in *myo1Δ *a background

The *SLT2 *gene disruption in the YJR13 pRS316-*MYO1 *strain was performed by replacing the *SLT2 *gene with a *KanMX4 *module by homologous recombination using a PCR based method [32]. The *myo1Δ slt2Δ *pRS316-*MYO1 *strain was grown in CSM 1 mg/ml 5-Fluoroorotic acid (CSM 5-FOA) to extract the plasmid and obtain a *myo1Δslt2Δ *double mutant.

### Western blot analysis of phosphorylated Slt2p levels

Wild type, *myo1Δ*, wild type pRS316-*RPL30*, wild type pRS316-*RPS31*, *myo1Δ *pRS316-*RPL30*, and *myo1Δ *pRS316-*RPS31 *yeast strains were grown in selective medium in 2% glucose or 2% galactose to an OD_600 _0.5–0.8 at 26°C, overnight. Cells were centrifuged for 5 minutes, washed in ice cold medium, and resuspended in lysis buffer as described in [24]. Protein extract were centrifuged at 13,000 for 10 minutes at 4°C, the supernatant was removed and quantified using the DC Protein Assay method (BioRad). Total protein extracts (75 μg) were separated in a 10% SDS-PAGE gel and transferred to a nitrocellulose membrane at 30 V for 16 h at 4°C. Membrane was incubated with anti-phospho-p42/44 MAP kinase monoclonal antibody (1:2000) (Cell Signaling Technology) to detect phosphorylated Slt2p levels. Membrane was stripped and reprobed with a mouse monoclonal antibody against Pgk1p (1:125 dilution) (Molecular Probes, Invitrogen) as loading control.

## Authors' contributions

JFRQ participated in the data analysis, real time RT-PCR experiments, genetic knockout experiments, Western blot experiments, and writing of the manuscript. DGG and FERM participated in the data analysis. NDB performed Nikkomycin Z dosage suppressor screening, identification and characterization. RAI participated in the design of experiments, supervised data analysis, and provided expertise in DNA microarrays data processing and statistical analysis software. JRRM participated in the design of experiments, data analysis, interpretation of results, and writing of the manuscript.

## Supplementary Material

Additional File 1 A text table of differentially expressed genes (p-value ≤ 0.01) in *myo1Δ *strains divided according to biological process. All differentially expressed genes are included except those with unknown biological process. The list of genes with unknown biological process is available at Gene Expression Omnibus on the NCBI website (GSE5931). Click here for file
