# Supplementary material for: Global mRNA expression analysis in myosin II deficient strains of Saccharomyces cerevisiae reveals an impairment of cell integrity functions
Source: BMC Genomics. 2008 Jan 23;9:34. doi: 10.1186/1471-2164-9-34 (PMC2253530; doi:10.1186/1471-2164-9-34)
Supplement: Additional File 1 — A text table of differentially expressed genes (p-value ≤ 0.01) in myo1Δ strains divided according to biological process. All differentially expressed genes are included except those with unknown biological process. The list of genes with unknown biological process is available at Gene Expression Omnibus on the NCBI website (GSE5931). [file 1471-2164-9-34-S1.pdf]

YORF	Gene	Molecular Function	Fold change
**Carbohydrate metabolism**			
YAL017W	*PSK1*	- protein serine/threonine kinase activity	1.6
YGL227W	*VID30*	- molecular_function unknown	1.7
YJL155C	*FBP26*	- fructose-2,6-bisphosphate 2-phosphatase activity	2.2
YBR105C	*VID24*	- molecular_function unknown	2.4
YGR143W	*SKN1*	- glucosidase activity	2.4
YLR174W	*IDP2*	- isocitrate dehydrogenase NADP+ activity	2.6
YMR135C	*GID8*	- molecular_function unknown	2.8
YGL156W	*AMS1*	- alpha-mannosidase activity	3.1
YPR026W	*ATH1*	- alpha,alpha-trehalase activity	3.5
YIL155C	*GUT2*	- glycerol-3-phosphate dehydrogenase activity	3.8
YHR104W	*GRE3*	- aldehyde reductase activity<br>- aldo-keto reductase activity	4.1
YKR058W	*GLG1*	- glycogenin glucosyltransferase activity	4.6
YBR126C	*TPS1*	- alpha,alpha-trehalose-phosphate synthase UDP-forming activity	5.3
YDR001C	*NTH1*	- alpha,alpha-trehalase activity	5.4
YCL040W	*GLK1*	- glucokinase activity	6.82
YPR184W	*GDB1*	- 4-alpha-glucanotransferase activity<br>- amylo-alpha-1,6-glucosidase activity	7.3
YEL011W	*GLC3*	- 1,4-alpha-glucan branching enzyme activity	7.4
YDR074W	*TPS2*	- trehalose-phosphatase activity	7.5
YKL035W	*UGP1*	- UTP-glucose-1-phosphate uridylyltransferase activity	9.1
YGR256W	*GND2*	- phosphogluconate dehydrogenase decarboxylating activity	9.9
YFR053C	*HXK1*	- hexokinase activity	14.2
YBR117C	*TKL2*	- transketolase activity	14.3
YPR160W	*GPH1*	- glycogen phosphorylase activity	18.9
Down regulated			
YOL056W	*GPM3*	- NONE	1.5
YER120W	*SCS2*	- protein binding	1.6
YER003C	*PMI40*	- mannose-6-phosphate isomerase activity	2.0
YHR163W	*SOL3*	- 6-phosphogluconolactonase activity	2.1
YDL103C	*QRI1*	- UDP-N-acetylglucosamine diphosphorylase activity	2.7
YKL127W	*PGM1*	- phosphoglucomutase activity	3.2
YDL055C	*PSA1*	- mannose-1-phosphate guanylyltransferase	3.5

**YORF**	**Gene**	**Molecular Function**	**Fold change**
		activity	
**protein amino acid phosphorylation**			
YJL141C	*YAK1*	- protein kinase activity	2.5
YLL019C	*KNS1*	- protein serine/threonine kinase activity<br>- protein-tyrosine kinase activity	2.8
Down regulated			
YGR123C	*PPT1*	- protein serine/threonine phosphatase activity	2.0
**signal transduction**			
YOL081W	*IRA2*	- Ras GTPase activator activity	3.7
YGL248W	*PDE1*	- cAMP-specific phosphodiesterase activity	2.5
YDR085C	*AFR1*	- receptor signaling protein activity	3.7
YOR134W	*BAG7*	- signal transducer activity<br>- Rho GTPase activator activity	1.9
YNL027W	*CRZ1*	- transcription factor activity	1.9
YNL173C	*MDG1*	- molecular_function unknown	2.1
YJL005W	*CYR1*	- adenylate cyclase activity	2.1
Down regulated			
YOR101W	*RAS1*	- RAS small monomeric GTPase activity	2.2
**budding**			
YDL188C	*PPH22*	- protein phosphatase type 2A activity	1.7
YER008C	*SEC3*	- molecular_function unknown	1.6
YGR070W	*ROM1*	- signal transducer activity<br>- Rho guanyl-nucleotide exchange factor activity	3.2
YDR129C	*SAC6*	- actin cable sensu Saccharomyces<br>- actin cortical patch sensu Saccharomyces	1.8
Down regulated			
YOR198C	*BFR1*	- RNA binding	2.4
YDR309C	*GIC2*	- small GTPase regulatory/interacting protein activity	2.4
YOR326W	*MYO2*	- microfilament motor activity	1.5
YDR507C	*GIN4*	- protein kinase activity	2.2
YHR023W	*MYO1*	- microfilament motor activity	4.5
**Transport**			
YJR121W	*ATP2*	- hydrogen-transporting ATP synthase activity, rotational mechanism	2.8
YJR059W	*PTK2*	- protein kinase activity	2.9
YPL134C	*ODC1*	- intracellular transporter activity	1.9

YORF	Gene	Molecular Function	Fold change
		- organic acid transporter activity	
YDL234C	*GYP7*	- Rab GTPase activator activity	1.9
YKL188C	*PXA2*	- ATP-binding cassette ABC transporter activity	2.5
YDR276C	*PMP3*	- molecular_function unknown	2.6
YLR093C	*NYV1*	- v-SNARE activity	1.9
YGL006W	*PMC1*	- calcium-transporting ATPase activity	2..3
YLL043W	*FPS1*	- transporter activity<br>- glycerol transporter activity	3.0
Down regulated			
YBR106W	*PHO88*	- phosphate transporter activity	3.2
YOR216C	*RUD3*	- molecular_function unknown	2.4
YCR034W	*FEN1*	- fatty acid elongase activity	2.9
YLR188W	*MDL1*	- ATP-binding cassette ABC transporter activity<br>- peptide-transporting ATPase activity<br>- ATPase activity	1.9
YDL192W	*ARF1*	- ARF small monomeric GTPase activity	1.9
YMR241W	*YHM2*	- tricarboxylate carrier activity	2.7
YJL145W	*SFH5*	- phosphatidylinositol transporter activity	2.5
YGR172C	*YIP1*	- molecular_function unknown	1.7
YHR175W	*CTR2*	- copper ion transporter activity<br>- copper uptake transporter activity	1.9
YNL231C	*PDR16*	- phosphatidylinositol transporter activity	1.9
YOL158C	*ENB1*	- ferric-enterobactin transporter activity	1.8
YAL022C	*FUN26*	- nucleoside transporter activity	1.8
YAL007C	*ERP2*	- molecular_function unknown	2.5
YDR534C	*FIT1*	- molecular_function unknown	4.6
YDR170C	*SEC7*	- ARF guanyl-nucleotide exchange factor activity	1.5
**Cell organization and biogenesis**			
YHR030C	*SLT2*	- MAP kinase activity	2.1
YMR053C	*STB2*	- transcriptional repressor activity	1.7
YKR076W	*ECM4*	- molecular_function unknown	3.2
YDR077W	*SED1*	- structural constituent of cell wall	3.7
YOL004W	*SIN3*	- histone deacetylase activity	2.3
YOR191W	*RIS1*	- DNA dependent ATPase activity	1.6
YGR282C	*BGL2*	- glucan 1,3-beta-glucosidase activity	2.2
YDR358W	*GGA1*	- molecular_function unknown	3.4
YMR302C	*PRP12*	- exonuclease activity	2..3
YDL223C	*HBT1*	- molecular_function unknown	7.3

YORF	Gene	Molecular Function	Fold change
YIL033C	*BCY1*	- cAMP-dependent protein kinase inhibitor activity	2.0
YJL164C	*TPK1*	- protein serine/threonine kinase activity<br>- cAMP-dependent protein kinase activity	2.5
YJL100W	*LSB6*	- 1-phosphatidylinositol 4-kinase activity	1.7
YDR202C	*RAV2*	- molecular_function unknown	2.0
YJL042W	*MHP1*	- structural constituent of cytoskeleton	2.7
YLL040C	*VPS13*	- molecular_function unknown	2.2
YDL024C	*DIA3*	- acid phosphatase activity	3.9
Down regulated genes			
YJR135W-A	*TIM8*	- protein transporter activity	2.5
YDL014W	*NOP1*	- methyltransferase activity	3.1
YOL077C	*BRX1*	- 5S rRNA binding<br>- rRNA primary transcript binding	2.2
YPL234C	*TFP3*	- hydrogen-transporting ATPase activity, rotational mechanism	1.6
YFL004W	*VTC2*	- molecular_function unknown	1.8
YNL066W	*SUN4*	- glucosidase activity	6.6
YBR048W	*RPS11B*	- structural constituent of ribosome	4.0
YDR060W	*MAK21*	- molecular_function unknown	2.7
YDR418W	*RPL12B*	- structural constituent of ribosome	3.3
YNL030W	*HHF2*	- DNA binding	1.8
YDR025W	*RPS11A*	- structural constituent of ribosome	3.2
YER118C	*SHO1*	- osmosensor activity	1.7
YLR197W	*SIK1*	- molecular_function unknown	2.8
YCL059C	*KRR1*	- molecular_function unknown	2.4
YNR027W	*BUD17*	- molecular_function unknown	1.9
YPL131W	*RPL5*	- RNA binding<br>- structural constituent of ribosome	2.4
YMR307W	*GAS1*	- 1,3-beta-glucanosyltransferase activity	2.9
YML073C	*RPL6A*	- RNA binding<br>- structural constituent of ribosome	2.5
YML069W	*POB3*	- chromatin binding	1.8
YLR167W	*RPS31*	- structural constituent of ribosome<br>- ribosomal chaperone activity	2.6
YKL185W	*ASH1*	- specific transcriptional repressor activity	2.1
YML061C	*PIF1*	- DNA helicase activity	2.1
YDL226C	*GCS1*	- ARF GTPase activator activity<br>- actin binding	1.9
YGR214W	*RPS0A*	- structural constituent of ribosome	2.7
YDL198C	*YHM1*	- transporter activity	3.8
YGL100W	*SEH1*	- structural molecule activity	1.6

YORF	Gene	Molecular Function	Fold change
YGL099W	*LSG1*	- GTPase activity	2.2
YGL097W	*SRM1*	- signal transducer activity	1.9
YJL158C	*CIS3*	- structural constituent of cell wall	4.2
YBL032W	*HEK2*	- mRNA binding	1.6
YNL283C	*WSC2*	- transmembrane receptor activity	2.0
YBL003C	*HTA2*	- DNA binding	6.5
YBL002W	*HTB2*	- DNA binding	3.7
YBR245C	ISW1	- ATPase activity	1.7
YER006W	*NUG1*	- GTPase activity	2.0
YDL143W	*CCT4*	- chaperone activity	1.7
YHR170W	*NMD3*	- RNA binding<br>- protein binding	2.2
YPR102C	*RPL11A*	- structural constituent of ribosome	2.8
YGL030W	*RPL30*	- structural constituent of ribosome	3.1
YEL054C	*RPL12A*	- structural constituent of ribosome	2.6
YDR225W	*HTA1*	- DNA binding	5.9
YDR224C	*HTB1*	- DNA binding	2.4
YLR409C	*UTP21*	- snoRNA binding	2.2
YEL040W	*UTR2*	- molecular_function unknown	4.1
YGR108W	*CLB1*	- cyclin-dependent protein kinase regulator activity	3.2
YNL188W	*KAR1*	- protein binding	1.5
YHR108W	*GGA2*	- molecular_function unknown	2.4
YAL025C	*MAK16*	- molecular_function unknown	3.3
YGR078C	*PAC10*	- tubulin binding	2.0
YOL127W	*RPL25*	- RNA binding<br>- structural constituent of ribosome	2.9
YKL013C	*ARC19*	- structural molecule activity	1.6
YKL009W	*MRT4*	- molecular_function unknown	3.6
YLR353W	*BUD8*	- molecular_function unknown	1.5
YNL153C	*GIM3*	- tubulin binding	2.1
YNL142W	*MEP2*	- ammonium transporter activity	3.2
YGR041W	*BUD9*	- molecular_function unknown	3.0
YHR062C	*RPP1*	- ribonuclease P activity<br>- ribonuclease MRP activity	1.8
YJR145C	*RPS4A*	- structural constituent of ribosome	2.8
YJR144W	*MGM101*	- DNA binding	1.8
**Stress response**			
YDR513W	*TTR1*	- thiol-disulfide exchange intermediate activity<br>- glutathione peroxidase activity<br>- glutathione transferase activity	1.9

YORF	Gene	Molecular Function	Fold change
YNL160W	*YGP1*	- molecular_function unknown	7.6
YGR088W	*CTT1*	- catalase activity	26.9
YDR214W	*AHA1*	- chaperone activator activity	3.8
YMR169C	*ALD3*	- aldehyde dehydrogenase activity	41.5
YMR250W	*GAD1*	- glutamate decarboxylase activity	7.1
YMR251W-A	*HOR7*	- molecular_function unknown	9.2
YKL150W	*MCR1*	- cytochrome-b5 reductase activity	6.1
YML070W	*DAK1*	- glycerone kinase activity	5.9
YIL101C	*XBP1*	- transcription factor activity	2.3
YKR066C	*CCP1*	- cytochrome-c peroxidase activity	7.0
YKR072C	*SIS2*	- protein phosphatase inhibitor activity<br>- phosphopantothenoylcysteine decarboxylase activity	1.9
YPL223C	*GRE1*	- molecular_function unknown	9.5
YBR072W	*HSP26*	- heat shock protein activity<br>- chaperone activity	23.3
YOL052C-A	*DDR2*	- molecular_function unknown	3.6
YFL014W	*HSP12*	- heat shock protein activity	36.7
YGR008C	*STF2*	- molecular_function unknown	7.3
Down regulated			
YIL053W	*RHR2*	DL-glycerol-3-phosphatase	2.7
YFL010C	*WWM1*	WW domain containing protein interacting with Metacaspase MCA1	1.8
**DNA metabolism**			
YIR002C	*MPH1*	- RNA helicase activity	1.9
YMR173W	*DDR48*	- molecular_function unknown	4.1
YBR214W	*SDS24*	- molecular_function unknown	2.6
YER116C	*SLX8*	- DNA binding	1.9
YML128C	*MSC1*	- molecular_function unknown	2.1
Down regulated			
YDR097C	*MSH6*	- ATPase activity<br>- ATP binding<br>- DNA binding	2.2
YER171W	*RAD3*	- general RNA polymerase II transcription factor activity<br>- DNA helicase activity	1.8
YJR043C	*POL32*	- delta DNA polymerase activity	2.1
YDL227C	*HO*	- endonuclease activity	4.1
YER070W	*RNR1*	- ribonucleoside-diphosphate reductase activity	4.6
YNL312W	*RFA2*	- DNA binding	2.0

YORF	Gene	Molecular Function	Fold change
YBL035C	*POL12*	- alpha DNA polymerase activity	2.4
YBL023C	*MCM2*	- chromatin binding<br>- ATP dependent DNA helicase activity	1.9
YOR074C	*CDC21*	- thymidylate synthase activity	3.1
YKL113C	*RAD27*	- 5 flap endonuclease activity	2.1
YHR154W	*RTT107*	- molecular_function unknown	2.1
YDL102W	*CDC2*	- delta DNA polymerase activity	1.8
**Protein transport**			
YLR259C	*HSP60*	- heat shock protein activity	2.9
YHL002W	*HSE1*	- protein binding	1.6
YBL075C	*SSA3*	- heat shock protein activity	3.7
YGL104C	*VPS73*	- molecular_function unknown	1.8
YDR200C	*VPS64*	- molecular_function unknown	1.8
YKL034W	*TUL1*	- ligase activity	1.9
Down regulated			
YGR082W	*TOM20*	- protein transporter activity	1.8
YDR372C	*VPS74*	- molecular_function unknown	1.8
YLR292C	*SEC72*	- protein transporter activity	2.0
YFL045C	*SEC53*	- phosphomannomutase activity	2.6
**Metabolism**			
YDR530C	*APA2*	- bis 5 -nucleosyl -tetraphosphatase asymmetrical activity<br>- bis 5 -nucleosyl -tetraphosphatase activity	1.7
YBR169C	*SSE2*	- heat shock protein activity	7.1
YFR033C	*QCR6*	- ubiquinol-cytochrome-c reductase activity	2.6
YBR183W	*YPC1*	- ceramidase activity	2.9
YEL039C	*CYC7*	- electron carrier activity	6.7
YOR377W	*ATF1*	- alcohol O-acetyltransferase activity	2.2
YAL060W	*BDH1*	- R,R -butanediol dehydrogenase activity	5.9
YDR231C	*COX20*	- chaperone activity	2.2
YOR020C	*HSP10*	- chaperone activity	2.3
YML004C	*GLO1*	- lactoylglutathione lyase activity	6.4
YBL015W	*ACH1*	- acetyl-CoA hydrolase activity	2.1
YLR120C	*YPS1*	- aspartic-type endopeptidase activity	1.9
YJL166W	*QCR8*	- ubiquinol-cytochrome-c reductase activity	2.7
YBL045C	*COR1*	- ubiquinol-cytochrome-c reductase activity	2.7
YDL205C	*HEM3*	- hydroxymethylbilane synthase activity	2.2
YML042W	*CAT2*	- carnitine O-acetyltransferase activity	3.9
YML054C	*CYB2*	- L-lactate dehydrogenase cytochrome activity	5.4
YPR191W	*QCR2*	- ubiquinol-cytochrome-c reductase activity	3.1
YMR304W	*UBP15*	- ubiquitin-specific protease activity	1.8

YORF	Gene	Molecular Function	Fold change
YNR019W	*ARE2*	- sterol O-acyltransferase activity	1.7
YDR034C	*LYS14*	- transcriptional activator activity	1.8
YML120C	*NDI1*	- oxidoreductase activity, acting on NADH or NADPH, quinone or similar compound as acceptor	7.5
YER141W	*COX15*	- molecular_function unknown	2.5
YLR228C	*ECM22*	- RNA polymerase II transcription factor activity	1.7
YOR219C	*STE13*	- aminopeptidase activity	1.6
Down regulated			
YLR303W	*MET17*	- O-acetylhomoserine aminocarboxypropyltransferase activity<br>- cysteine synthase activity	5.4
YDR465C	*RMT2*	- protein-arginine N-methyltransferase activity	2.4
YOR241W	*MET7*	- tetrahydrofolylpolyglutamate synthase activity	1.9
YOR236W	*DFR1*	- dihydrofolate reductase activity	2.8
YCR053W	*THR4*	- threonine synthase activity	2.4
YGL213C	*SKI8*	- translation repressor activity	1.7
YML126C	*ERG13*	- hydroxymethylglutaryl-CoA synthase activity	2.2
YML106W	*URA5*	- orotate phosphoribosyltransferase activity	3.5
YGR285C	*ZUO1*	- chaperone activity	1.8
YDR380W	*ARO10*	- carboxy-lyase activity<br>- pyruvate decarboxylase activity<br>- phenylpyruvate decarboxylase activity	3.6
YKL211C	*TRP3*	- anthranilate synthase activity<br>- indole-3-glycerol-phosphate synthase activity	1.9
YGL148W	*ARO2*	- chorismate synthase activity	2.6
YML075C	*HMG1*	- hydroxymethylglutaryl-CoA reductase NADPH activity	1.6
YMR300C	*ADE4*	- amidophosphoribosyltransferase activity	2.0
YLR153C	*ACS2*	- acetate-CoA ligase activity	2.3
YMR274C	*RCE1*	- prenyl-dependent CAAX protease activity	1.9
YDR321W	*ASP1*	- asparaginase activity	2.4
YNL315C	*ATP11*	- chaperone activity	1.6
YER055C	*HIS1*	- ATP phosphoribosyltransferase activity	2.5
YER043C	*SAH1*	- adenosylhomocysteinase activity	3.1
YDR300C	*PRO1*	- glutamate 5-kinase activity	1.7
YPR145W	*ASN1*	- asparagine synthase glutamine-	4.1

YORF	Gene	Molecular Function	Fold change
		hydrolyzing activity	
YML008C	*ERG6*	- sterol 24-C-methyltransferase activity	2.7
YER023W	*PRO3*	- pyrroline-5-carboxylate reductase activity	2.1
YLR100W	*ERG27*	- 3-keto sterol reductase activity	2.6
YBR252W	*DUT1*	- dUTP diphosphatase activity	2.4
YBR249C	*ARO4*	- 3-deoxy-7-phosphoheptulonate synthase activity	2.8
YBR248C	*HIS7*	- imidazoleglycerol phosphate synthase activity	2.0
YMR202W	*ERG2*	- C-8 sterol isomerase activity	4.0
YDL141W	*BPL1*	- biotin-[acetyl-CoA-carboxylase] ligase activity<br>- biotin-[methylcrotonoyl-CoA-carboxylase] ligase activity<br>- biotin-[methylmalonyl-CoA-carboxytransferase] ligase activity<br>- biotin-[propionyl-CoA-carboxylase ATP-hydrolyzing] ligase activity	2.1
YLR066W	*SPC3*	- signal peptidase activity	2.2
YLR432W	*IMD3*	- IMP dehydrogenase activity	2.2
YGL012W	*ERG4*	- delta24 24-1 sterol reductase activity	2.5
YHR128W	*FUR1*	- uracil phosphoribosyltransferase activity	3.3
YHR123W	*EPT1*	- ethanolaminephosphotransferase activity	1.8
YLR017W	*MEU1*	- molecular_function unknown	1.7
YKL008C	*LAC1*	- sphingosine N-acyltransferase activity	1.9
YDR518W	*EUG1*	- protein disulfide isomerase activity	2.2
YNL130C	*CPT1*	- diacylglycerol cholinephosphotransferase activity	1.8
**Sporulation**			
YLL039C	*UBI4*	- ATP-dependent protein binding	6.5
YLR028C	*ADE16*	- IMP cyclohydrolase activity<br>- phosphoribosylaminoimidazolecarboxamide formyltransferase activity	1.9
YNL202W	*SPS19*	- 2,4-dienoyl-CoA reductase NADPH activity	3.3
YHR139C	*SPS100*	- molecular_function unknown	6.9
Down regulated			
YJL074C	*SMC3*	- ATPase activity	2.4
YIL026C	*IRR1*	- nuclear cohesin complex	2.6
**Cell cycle**			

**YORF**	**Gene**	**Molecular Function**	**Fold change**
YFR040W	*SAP155*	- protein serine/threonine phosphatase activity	2.5
YMR165C	*SMP2*	- molecular_function unknown	2.4
YML034W	*SRC1*	- molecular_function unknown	2.5
Down regulated			
YBR158W	*AMN1*	- protein binding	2.2
YAL034W-A	*MTW1*	- molecular_function unknown	1.7
YGL021W	*ALK1*	- protein serine/threonine kinase activity	1.8
YDL003W	*MCD1*	- molecular_function unknown	2.8
YNL289W	*PCL1*	- cyclin-dependent protein kinase regulator activity	3.2
**Autophagy**			
YPL154C	*PEP4*	- saccharopepsin activity<br>- endopeptidase activity	3.2
YNL223W	*ATG4*	- microtubule binding	1.6
Down regulated			
**RNA processing**			
YNR034W	*SOL1*	- 6-phosphogluconolactonase activity	2.3
YLR107W	*REX3*	- 3 -5 exonuclease activity	1.9
YGR178C	*PBP1*<br>*MRS16*	- molecular_function unknown	1.7
YGR129W	*SYF2*	- pre-mRNA splicing factor activity	2.1
Down regulated			
YFL001W	*DEG1*	- pseudouridylate synthase activity	1.5
YGL120C	*PRP43*	- ATP dependent RNA helicase activity<br>- pre-mRNA splicing factor activity	2.6
YER032W	*FIR1*	- molecular_function unknown	1.8
YAR008W	*SEN34*	- tRNA-intron endonuclease activity	2.6
YDL051W	*LHP1*	- RNA binding	2.2
**protein amino acid dephosphorylation**			
YER054C	*GIP2*	- protein phosphatase regulator activity	3.9
**Transcription**			
YIL038C	*NOT3*	- 3 -5 exoribonuclease activity	1.6
Down regulated			
YPR187W	*RPO26*	- DNA-directed RNA polymerase activity	2.2

YORF	Gene	Molecular Function	Fold change
YOR340C	*RPA43*	- DNA-directed RNA polymerase activity	2.2
YDL150W	*RPC53*	- DNA-directed RNA polymerase activity	1.8
YOL005C	*RPB11*	- DNA-directed RNA polymerase activity	2.0
YDR451C	*YHP1*	- specific RNA polymerase II transcription factor activity	2.8
**Cell growth and maintainance**			
YDR436W	*PPZ2*	- protein serine/threonine phosphatase activity	1.7
Down regulated			
YGL028C	*SCW11*	- glucan 1,3-beta-glucosidase activity	2.3
**Protein byosynthesis**			
YOR317W	*FAA1*	- long-chain-fatty-acid-CoA ligase activity	3.0
YKL201C	*MNN4*	- molecular_function unknown	2.5
Down regulated			
YOR276W	*CAF20*	- translation regulator activity	2.1
YJR123W	*RPS5*	- structural constituent of ribosome	2.9
YFL022C	*FRS2*	- phenylalanine-tRNA ligase activity	1.9
YPL249C-A	*RPL36B*	- RNA binding<br>- structural constituent of ribosome	2.3
YNL096C	*RPS7B*	- structural constituent of ribosome	2.1
YDR471W	*RPL27B*	- structural constituent of ribosome	2.6
YBR084W	*MIS1*	- formate-tetrahydrofolate ligase activity<br>- methenyltetrahydrofolate cyclohydrolase activity<br>- methylenetetrahydrofolate dehydrogenase NADP+ activity	2.3
YGL257C	*MNT2*	- alpha-1,3-mannosyltransferase activity	2.4
YOL040C	*RPS15*	- structural constituent of ribosome	2.5
YPL220W	*RPL1A*	- structural constituent of ribosome	2.5
YNL069C	*RPL16B*	- RNA binding<br>- structural constituent of ribosome	1.9
YNL067W	*RPL9B*	- structural constituent of ribosome	2.4
YKR057W	*RPS21A*	- structural constituent of ribosome	2.3
YHL033C	*RPL8A*	- structural constituent of ribosome	3.2
YIL133C	*RPL16A*	- RNA binding<br>- structural constituent of ribosome	2.7
YCR046C	*IMG1*	- structural constituent of ribosome	1.6
YLR249W	*YEF3*	- translation elongation factor activity	2.3

YORF	Gene	Molecular Function	Fold change
YKR026C	*GCN3*	- translation initiation factor activity	1.9
YDR037W	*KRS1*	- lysine-tRNA ligase activity	2.5
YGL189C	*RPS26A*	- structural constituent of ribosome	2.1
YER131W	*RPS26B*	- structural constituent of ribosome	2.8
YPL143W	*RPL33A*	- structural constituent of ribosome	1.9
YGR264C	*MES1*	- methionine-tRNA ligase activity	2.0
YER102W	*RPS8B*	- structural constituent of ribosome	2.8
YGL147C	*RPL9A*	- structural constituent of ribosome	2.4
YIL052C	*RPL34B*	- structural constituent of ribosome	2.1
YGL135W	*RPL1B*	- structural constituent of ribosome	2.7
YML063W	*RPS1B*	- structural constituent of ribosome	3.3
YKL180W	*RPL17A*	- structural constituent of ribosome	3.5
YER074W	*RPS24A*	- structural constituent of ribosome	2.1
YJL190C	*RPS22A*	- structural constituent of ribosome	3.3
YGL123W	*RPS2*	- structural constituent of ribosome	2.4
YPL090C	*RPS6A*	- structural constituent of ribosome	2.5
YIL018W	*RPL2B*	- structural constituent of ribosome	3.3
YJL177W	*RPL17B*	- structural constituent of ribosome	4.8
YER056C-A	*RPL34A*	- structural constituent of ribosome	2.9
YPL079W	*RPL21B*	- structural constituent of ribosome	3.2
YOR096W	*RPS7A*	- structural constituent of ribosome	2.6
YDL184C	*RPL41A*	- structural constituent of ribosome	1.9
YGR185C	*TYS1*	- tyrosine-tRNA ligase activity	1.6
YGL076C	*RPL7A*	- structural constituent of ribosome	2.0
YHR203C	*RPS4B*	- structural constituent of ribosome	2.7
YHR193C	*EGD2*	- chaperone activity	2.1
YDL136W	*RPL35B*	- structural constituent of ribosome	2.2
YDL133C-A	*RPL41B*	- structural constituent of ribosome	2.1
YGL031C	*RPL24A*	- RNA binding<br>- structural constituent of ribosome	3.2
YLR061W	*RPL22A*	- structural constituent of ribosome	3.9
YKL081W	*TEF4*	- translation elongation factor activity	2.9
YLR060W	*FRS1*	- phenylalanine-tRNA ligase activity	2.5
YGL022W	*STT3*	- dolichyl-diphosphooligosaccharide-protein glycotransferase activity	1.8
YEL031W	*SPF1*	- ATPase activity, coupled to transmembrane movement of ions, phosphorylative mechanism	2.3
YBR191W	*RPL21A*	- structural constituent of ribosome	3.8
YBR189W	*RPS9B*	- structural constituent of ribosome	2.4
YLR388W	*RPS29A*	- structural constituent of ribosome	2.0
YBR181C	*RPS6B*	- structural constituent of ribosome	2.5
YDL083C	*RPS16B*	- structural constituent of ribosome	1.9

**YORF**	**Gene**	**Molecular Function**	**Fold change**
YDL082W	*RPL13A*	- structural constituent of ribosome	3.2
YMR142C	*RPL13B*	- structural constituent of ribosome	2.2
YFR032C-A	*RPL29*	- structural constituent of ribosome	2.1
YPR043W	*RPL43A*	- structural constituent of ribosome	2.8
YOL139C	*CDC33*	- translation initiation factor activity	2.1
YFR031C-A	*RPL2A*	- structural constituent of ribosome	3.6
YDL061C	*RPS29B*	- structural constituent of ribosome	2.0
YOL121C	*RPS19A*	- structural constituent of ribosome	2.1
YOL120C	*RPL18A*	- structural constituent of ribosome	3.7
YNL162W	*RPL42A*	- structural constituent of ribosome	2.4
YLL045C	*RPL8B*	- structural constituent of ribosome	2.6
YOR312C	*RPL20B*	- structural constituent of ribosome	3.3
YLR344W	*RPL26A*	- RNA binding<br>- structural constituent of ribosome	2.6
YOL097C	*WRS1*	- tryptophan-tRNA ligase activity	2.7
YBR121C	*GRS1*	- glycine-tRNA ligase activity	2.1
YJR143C	*PMT4*	- dolichyl-phosphate-mannose-protein mannosyltransferase activity	3.1
YGR034W	*RPL26B*	- RNA binding<br>- structural constituent of ribosome	2.6
**DNA damage response**			
Down regulated			
YNL178W	*RPS3*	- structural constituent of ribosome	3.2
RNA localization			
Down regulated			
YJL080C	*SCP160*	- RNA binding	2.6
